# Factors relating to participation in follow-up to the 45 and up study in Aboriginal and non-Aboriginal individuals

**DOI:** 10.1186/s12874-016-0155-x

**Published:** 2016-05-11

**Authors:** Lina Gubhaju, Emily Banks, Rona Macniven, Grace Joshy, Bridgette J. McNamara, Adrian Bauman, Sandra J. Eades

**Affiliations:** Aboriginal Health, Baker IDI Heart and Diabetes Institute, 99 Commercial Road, Melbourne, 3004 Victoria Australia; National Centre for Epidemiology and Population Health, Research School of Population Health, Australian National University, Acton, 2601 Australian Capital Territory Australia; Prevention Research Collaboration, Sydney School of Public Health, The University of Sydney, Level 6 The Hub, The Charles Perkins Centre (D17), Sydney, 2006 New South Wales Australia

**Keywords:** Aboriginal people, Cohort study, Loss-to-follow up, Participation rates, Socio-demographic factors, Health behaviours

## Abstract

**Background:**

This study aimed to characterise the factors relating to participation in a postal follow-up study in Aboriginal and non-Aboriginal individuals, given the need to quantify potential biases from loss to follow-up and the lack of evidence regarding postal surveys among Aboriginal people.

**Methods:**

The first 100,000 participants from the Sax Institute’s 45 and Up Study, a large scale cohort study, were posted a follow-up questionnaire gathering general demographic, health and risk factor data, emphasising Social, Economic and Environmental Factors (“The SEEF Study”). For each variable of interest, percentages of those invited who went on to participate in follow-up were tabulated separately for Aboriginal and non-Aboriginal participants and age- and sex-adjusted participation rate ratios (aPRR) were calculated.

**Results:**

Of the 692 Aboriginal and 97,178 non-Aboriginal invitees to the study, 314 Aboriginal (45 %) and 59,175 non-Aboriginal (61 %) individuals responded. While Aboriginal people were less likely to respond than non-Aboriginal people (aPRR 0.72, 95 % CI 0.66–0.78), factors related to response were similar. Follow-up study participants were more likely than non-participants to have university versus no educational qualifications (1.6, 1.3–2.0 [Aboriginal]; 1.5, 1.5–1.5 [non-Aboriginal]) and an annual income of ≥70,000 versus < $20,000 (1.6, 1.3–2.0; 1.2, 1.2–1.3 [*χ*^2^ = 7.7; *p* = 0.001]). Current smokers (0.55, 0.42–0.72; 0.76, 0.74–0.77 [*χ*^2^ = 7.14; *p* = 0.03]), those reporting poor self-rated health (0.68, 0.47–0.99; 0.65, 0.61–0.69), poor quality of life (0.63, 0.41–0.97; 0.61, 0.57–0.66) and very high psychological distress (0.71, 0.68–0.75 [non-Aboriginal]) were less likely than other cohort members to respond.

**Conclusions:**

Relatively large numbers of Aboriginal and non-Aboriginal individuals participated in the first 45 and Up Study follow-up suggesting that postal surveys can be used to follow Aboriginal participants in cohort studies. Despite somewhat greater loss to follow-up in Aboriginal people (after considering socio-demographic and health characteristics), factors related to follow-up participation were similar in both groups: greater loss was observed in those experiencing disadvantage, ill-health and health risk, with implications for interpretation of future findings. Aboriginal low income earners and current regular smokers had a particularly elevated likelihood of non-participation compared to non-Aboriginal people. These findings highlight the importance of identifying and addressing barriers to participation among hard-to-reach population groups.

**Electronic supplementary material:**

The online version of this article (doi:10.1186/s12874-016-0155-x) contains supplementary material, which is available to authorized users.

## Background

Prospective cohort studies have demonstrated their importance in providing reliable data on estimates of incidence and the relative risk of various outcomes in populations of interest. Despite their well-established use in epidemiology and population health, it is recognised that major differences in the characteristics of the participants who were initially studied at baseline from those who are followed-up can lead to bias [1, 2]. A sound quantitative understanding of patterns of non-response is important for interpreting longitudinal data from cohort studies, including whether such non-response is likely to materially affect findings.

It is well known that although postal surveys are a useful, convenient and cost-effective method of conducting longitudinal follow-up studies, particularly among geographically dispersed population groups [3], this method is prone to non-response [4]. Therefore, it is important to quantify participant characteristics associated with non-response in postal follow-up surveys in order to aid in interpretation of study findings and guide future studies. Willingness to participate in health research surveys among the general Australian population has recently been examined by Glass and colleagues (2015) [5]; they reported greater willingness among women versus men, older versus younger people and those with a long-term disease or disability. However, few studies to date have examined characteristics of participants lost to follow-up.

Loss to follow-up is likely to vary by population level characteristics. A systematic literature review of attrition between waves in longitudinal studies in the elderly due to reasons other than death reported that in general, people in worse health were less likely to be re-contactable [6]. The review also found that very few longitudinal studies actually report the risk factors for attrition. Among those that have reported such findings, social factors such as contact with friends/family and level of social support have not been examined as often as the basic socio-demographic factors such as education, income and marital status.

It has also often been reported that recruitment and retention of ethnic minorities in research studies is a challenge [7–9]. In Australia, due to the history of how Aboriginal health research had been previously conducted, including issues relating to ethical conduct, Aboriginal people are justified in viewing research studies negatively [10, 11]. It has also been widely speculated that retaining Aboriginal people in cohort studies, particularly through postal surveys is difficult given the highly mobile nature of the Aboriginal population in Australia [12]. Previous longitudinal studies have reported higher attrition rates among Aboriginal participants compared to non-Aboriginal participants [13–15]. However, to date there have been no studies that have examined the characteristics of responders versus non-responders in Aboriginal people and whether these differ between Aboriginal and non-Aboriginal people. Therefore, the aim of this study was to examine the socio-demographic and health-related characteristics of Aboriginal and non-Aboriginal participants in an initial postal follow-up of the 45 and Up study.

## Methods

The Sax Institute’s 45 and Up Study is a large scale longitudinal cohort study of men and women aged 45 years and older from the general population of New South Wales (NSW), Australia that has been designed to provide reliable evidence to inform policy to support healthy ageing. Further information about the study is available at http://www.45andup.org.au/ .

Details of participant recruitment and data collection have been reported previously [16]. Briefly, individuals aged 45 years and over were randomly selected from the Medicare Australia database (the national universal health insurance scheme), with a two-fold oversampling of rural areas and individuals aged 80 years and over. Participants entered the study by completing a baseline postal questionnaire which was distributed between 1 February 2006 to 31 December 2008 and providing written consent to follow their long term health, through repeat questionnaires and linkage to health records. A total of 267,153 people were recruited to the study over this time; the study had an overall 18 % response rate [16].

Aboriginal status was self-identified in the baseline questionnaire in response to the question: ‘Are you of Aboriginal or Torres Strait Islander Origin? With the following tick box options: 1) No 2) Yes, Aboriginal and 3) Yes, Torres Strait Islander; participants were able to select both Aboriginal and Torres Strait Islander. Of the total participants, 1949 people identified as being of Aboriginal and/or Torres Strait Islander origin. The study’s baseline questionnaire included a range of questions related to socio-demographic factors, physical and psychological health, behavioural risk factors, social support and past and present medical history. Baseline characteristics of Aboriginal and non-Aboriginal participants have been reported previously [17].

The initial follow-up to the 45 and Up study was undertaken from September-November 2010 where the first ~100,000 participants to join the 45 and Up Study were posted a questionnaire gathering general demographic, health and risk factor data, emphasising Social, Economic and Environmental Factors (as part of “The SEEF Study”). Participants who had requested not be contacted further, had already been contacted for other sub-studies or were deceased (ascertained through linkages to death registries) were not eligible for the follow-up study. A total of 99,927 participants of the 45 and Up study were invited to participate in the follow-up survey.

### Ethics, consent and permissions

The 45 and Up and SEEF studies as a whole have received ethical approval from the Human Research Ethics Committees of the University of New South Wales (reference 10186) and the University of Sydney (Ref No. 10-2009/12187), respectively. Ethical approval for the current study has been granted by the Aboriginal Health and Medical Research Council of New South Wales (912/13). All participants of this study provided written informed consent.

### Variables

All variables used in this study were derived from the self-reported 45 and Up baseline questionnaire apart from the Accessibility Remoteness Index of Australia Plus (ARIA+) score and the Index of Relative Socio-economic Disadvantage (IRSD) which were derived for each participant’s postcode of residence at the time of original recruitment as recorded by Medicare Australia. Australian Standard Geographical Classification (ASGC) Remoteness areas, based on enhanced measures of remoteness (ARIA+) developed by the National Key Centre for Social Applications of Geographic Information Systems, categorises areas as ‘major cities’, ‘inner regional’, ‘outer regional’, ‘remote’ and ‘very remote.’ The ARIA+ index values are based on road distance from a locality to the closest service centre [18]. IRSD is one of the four indexes in the Socio-Economic Indexes for Areas (SEIFA) and is primarily based on disadvantage and the variable is derived from census variables such as low income, low educational attainment, unemployment and dwellings without motor vehicles [19]. Socio-demographic information included age, sex, formal educational qualification, marital status, household annual pre-tax income, employment status, ARIA+ score and IRSD. Participants were grouped into quintiles of the IRSD score. Those in quintile 1 were the most disadvantaged and those in quintile 5 were the least disadvantaged [19].

Lifestyle and health risk factor variables included those relating to smoking, alcohol and body mass index (BMI), screen time, hours spent sitting, physical activity and diet. Self-reported weight and height measurements were used to calculate BMI, as weight in kilograms divided by the square of their height in metres (kg/m^2^). BMI was categorized according to the World Health Organization (WHO) criteria as underweight (BMI < 18.5 kg/m^2^), healthy weight (18.5–24.99 kg/m^2^), overweight (25.0–29.99 kg/m^2^) and obese (BMI ≥ 30 kg/m^2^) [20]. Participants’ overall level of physical activity was classified according to their responses to questions on the number of weekly sessions (of any duration) of moderate and vigorous physical activity and episodes of walking for longer than 10 min, using items from the validated Active Australia questionnaire [21]. A weighted weekly average number of sessions were calculated for each participant by adding the total number of sessions, with vigorous activity sessions receiving twice the weighting of moderate activity or walking sessions. Physical activity was classified as either ‘sufficient’ (150 min of physical activity in 5 or more sessions a week) or ‘insufficient’ (greater than 1 but less than or equal to 149 min), based on the guidelines from the Australian Institute of Health and Welfare [21]. Sedentary time was assessed based on ‘screen time’ which was the number of hours spent per day watching television or using the computer and ‘sitting time’ which was the number of hours per day spent sitting. Fruit (including fruit juice) and vegetable (including both raw and cooked vegetables) intake was assessed as servings per day and classified as adequate (≥2 servings of fruit and ≥ 5 servings of vegetables per day) or inadequate (less than these amounts) according to the National Health and Medical Research Council guidelines [22].

Self-rated health and quality of life were based on the question, “In general, how would you rate your: Overall health? Quality of life?” followed by options of excellent, very good, good, fair and poor. In order to determine the level of social support provided by close contacts, participants were asked “How many people outside your home, but within 1 h of travel, do you feel you can depend on or you feel very close to?” Based on the responses the social support variable was categorised as follows: none, 1–3 people, 4–6 people and 7 or more people. Psychological distress was measured using the Kessler-10 score [23]; a scale based on 10 items used to measure non-specific psychological distress. Logical imputations were performed for missing values where there is a valid value for a similar but more severe item. For example, when the value “how often did you feel: depressed” is missing, then the value for “how often did you feel: so depressed that nothing could cheer you up” is imputed to the less severe item. The average of all non-missing items is imputed for up to one missing item, and then the final score is calculated; a higher score indicated a higher level of psychological distress. Final scores were only calculated for those participants that had a response for all ten questions after imputation as described above. Kessler-10 scores were classified into 4 groups: low psychological distress (score 10–15), moderate psychological distress (score 16–21), high psychological distress (score 22–29) and very high psychological distress (score 30 or higher) [24].

Past history of and current treatment for certain medical conditions were assessed based on the participant’s response to the questions ‘Has the doctor ever told you that you have…’ and ‘In the last month have you been treated for…”, respectively, followed by a list of conditions that the participant could select. Categories of multiple morbidity were as follows: 0, 1–2 conditions, 3–4 conditions, 5 or more conditions.

Individuals who reported needing assistance with daily tasks because of long-term illness or disability were considered to have a major disability. Functional capacity was assessed using the Medical Outcomes Study Physical Functioning Scale [25]; a lower physical functioning score indicates more severe functional limitation [26]. The questions on the physical functioning scale asked whether participants are limited in their ability to perform vigorous and moderate physical activities and tasks such as: lifting shopping, climbing stairs, walking, bending, kneeling or stooping and bathing or dressing. A score is calculated where there are up to 5 missing items [25]. Functional limitation scores were classified into 5 groups: no limitation (score of 100), minor limitation (score 95–99), mild limitation (score 86–94), moderate limitation (60–84) and severe limitation (score 0–59).

### Statistical analyses

For each variable of interest, frequencies and percentages (expressed as a percentage of those invited to the study) were tabulated separately for Aboriginal and non-Aboriginal participants. Generalised linear models with a binomial distribution and a log link function (binomial regression) (proc genmod in SAS, v9.4) adjusted for age and sex was used to determine participation rate ratios (PRR) with participation in the follow-up (yes/no) as the outcome. To examine the mediating role of education and income, models were further adjusted for formal educational qualifications and household annual income level. Analyses were conducted separately in Aboriginal and in non-Aboriginal participants. Effect measure modification of the association between participation and each specific factor by Aboriginal status was assessed using likelihood-ratio tests which compare the age and sex adjusted model with and without the interaction term [27]. All statistical analyses were undertaken using SAS software version 9.4 (SAS Institute Inc, Cary, NC, USA).

## Results

### Overall response to the invitation to participate in the follow-up study

**Fig. 1:**
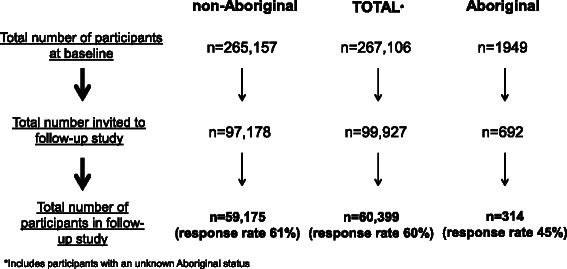
Number of Aboriginal and non-Aboriginal participants in the baseline 45 and Up study and follow-up study

A total of 99,927 participants (97,178 non-Aboriginal; 692 Aboriginal; 2057 unknown Aboriginal status) were invited to participate in the follow-up study of whom 60,399 responded (overall response rate = 60 %). Among Aboriginal people, 692 were invited, of whom 314 responded giving a response rate of 45 %. Among non-Aboriginal people 97,178 were invited, of whom 59,175 responded, giving a response rate of 61 % (Fig. 1). Participants without a valid Aboriginal status were excluded from the analyses. Aboriginal people were less likely to participate in the follow-up study compared to non-Aboriginal people; adjusting for age and sex the participation rate ratio was 0.72 (95 % CI 0.66–0.78). Following further adjustment for formal educational qualifications, annual household income and remoteness of residence, Aboriginal people remained significantly less likely to participate in the follow-up study compared to non-Aboriginal people (0.81, 0.74-0.87). Furthermore, after taking into account smoking status and number of medical conditions, Aboriginal people were still significantly less likely to participate in the follow-up study compared to non-Aboriginal people (0.83, 0.77-0.90).

### Response rate to the follow-up survey according to socio-demographic factors at baseline (Fig. 2)

Among both Aboriginal and non-Aboriginal people, females were marginally more likely to respond compared to males (49 % vs 41 % [Aboriginal] and 62 % vs 59 % [non-Aboriginal]). Participants between the ages of 50–69 at baseline were more likely to respond to the survey compared to those aged 45–49 years; this association strengthened among both Aboriginal and non-Aboriginal participants in the fully adjusted model (further adjustment for formal educational qualifications and household income level) (Additional file 1: Table S1). Rates of participation increased steadily with increasing levels of formal educational qualifications among both Aboriginal and non-Aboriginal people. Participants with a university degree or higher were more likely to participate compared to those with no educational qualifications (1.58, 1.25–2.01 [Aboriginal] and 1.49, 1.46–1.52 [non-Aboriginal]); the strength of this association was attenuated (1.24, 0.97–1.59 [Aboriginal] and 1.38, 1.35–1.41 [non-Aboriginal]) in the fully adjusted model (Additional file 1: Table S1). Unmarried/not-partnered participants were less likely to participate compared to those who were married or living with a partner; only about a third of Aboriginal participants who were single or widowed participated in the follow up survey. Participants who were disabled/sick/unemployed were significantly less likely to participate compared to those who were working full-time/part-time (0.72, 0.54–0.95 [Aboriginal] and 0.76, 0.74–0.79 [non-Aboriginal]); the strength of this association was attenuated in the fully adjusted model (0.95, 0.71–1.28 [Aboriginal] and 0.89, 0.87–0.92 [non-Aboriginal]) (Additional file 1: Table S1). Accordingly, those earning a higher annual household income were more likely to participate compared to those earning less than $20,000/year. The relationship between household income and participation persisted in the fully adjusted model, however the prevalence ratio was slightly attenuated (Additional file 1: Table S1). The association between annual household income and participation showed a statistically significant interaction with Aboriginal status (*χ*^2^ = 7.7; *P* = 0.001), suggesting that the relationship between household income and participation differed among Aboriginal people in comparison to non-Aboriginal people. Participants with a health care card were significantly less likely to participate compared to participants with private health insurance; the relationship between having private health insurance and participation differed according to Aboriginal status (*χ*^2^ = 7.92; *P* = 0.02). Participants living in inner regional areas were more likely to participate compared to those living in major cities. The relationship between remoteness of residence and participation also differed with Aboriginal status (*χ*^2^ = 14.3; *P* = 0.003). Aboriginal participants living in inner regional areas had a greater likelihood of participation compared to non-Aboriginal participants.

**Fig. 2:**
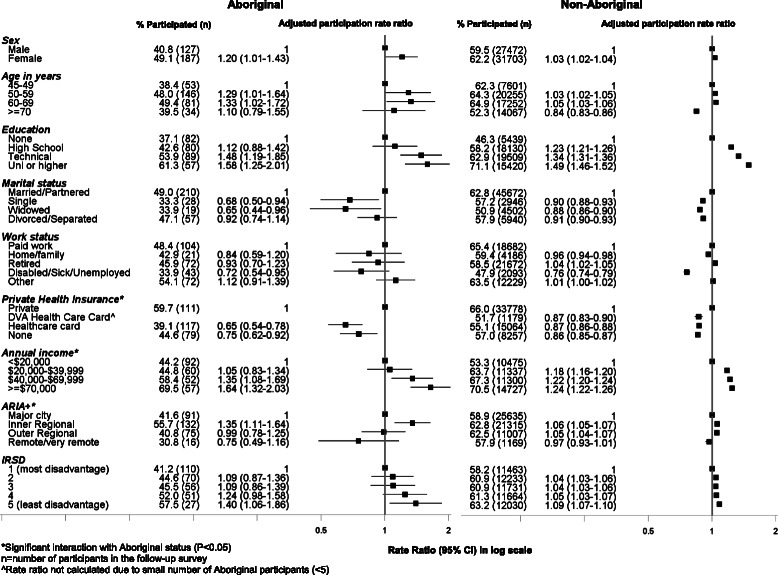
Follow-up participation in the 45 and Up Study among Aboriginal and non-Aboriginal individuals by socio-demographic characteristics at baseline

### Response rate to the follow-up survey according to health behaviours at baseline (Fig. 3)

Both Aboriginal and non-Aboriginal people who reported being regular smokers at baseline were significantly less likely to participate in the SEEF study compared to never smokers (0.55, 0.42–0.72 [Aboriginal]; 0.76, 0.74–0.77 [non-Aboriginal], respectively). This association persisted in the fully adjusted model (0.61, 0.47–0.79 [Aboriginal]; 0.81, 0.79–0.83 [non-Aboriginal) (Additional file 1: Table S2). The association between smoking status and participation showed a statistically significant interaction with Aboriginal status (*χ*^2^ = 7.14; *P* = 0.03). Aboriginal smokers were even less likely to participate compared to non-Aboriginal smokers. Among Aboriginal people, only 30 % of current smokers who were invited to the follow-up participated compared to 50 % among non-Aboriginal people. Those who consumed more than one alcoholic drink per week were more likely to participate in the follow-up study compared to those who did not consume alcohol. Compared to those who were normal weight, those who were underweight or obese were slightly less likely to participate in the study. Those people who did not meet the physical activity recommendations and had a poor diet (insufficient vegetable and fruit intake) were less likely to participate in the follow-up study. Those people who spent more time sitting (≥7 h) were marginally more likely to participate in the study compared to those who spent less time sitting (0–3 h).

**Fig. 3:**
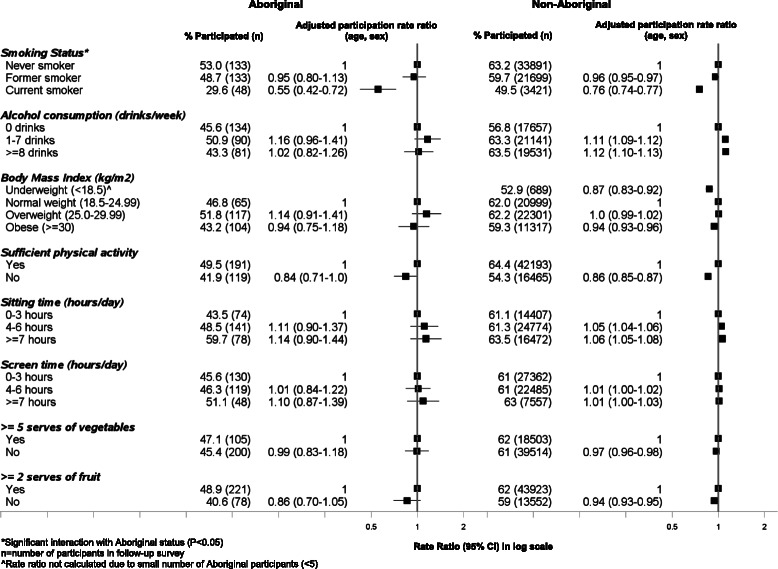
Follow-up participation in the 45 and Up Study among Aboriginal and non-Aboriginal individuals by health behaviours at baseline

### Response rate to the follow-up survey according to psychosocial factors at baseline (Fig. 4)

Psychosocial correlates of participation were very similar between Aboriginal and non-Aboriginal participants. Among both Aboriginal and non-Aboriginal groups, those people who had poor self-rated health were significantly less likely to participate compared to those who self-rated their health as excellent/very good (0.69, 0.47–0.99 [Aboriginal]; 0.65, 0.61–0.69 [non-Aboriginal]). Similarly, those participants with poor self-rated quality of life were also less likely to participate. Participation rate also decreased steadily with increasing levels of psychological distress. Among Aboriginal and non-Aboriginal people with high levels of psychological distress, 39 % and 46 % participated, respectively. The absolute and relative proportions of people participating in the follow up increased with increasing number of social contacts. Those individuals with full-time carer responsibilities were marginally less likely to participate compared to those with no carer responsibilities. The observed associations between psychosocial factors and participation in the follow-up survey among both Aboriginal and non-Aboriginal participants persisted in the fully adjusted model (Additional file 1: Table S3).

**Fig. 4:**
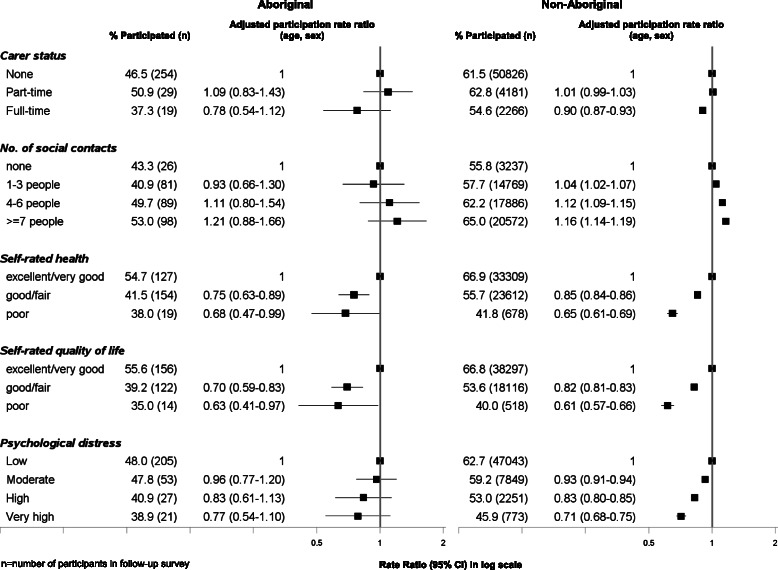
Follow-up participation in the 45 and Up Study among Aboriginal and non-Aboriginal individuals by psychosocial factors at baseline

### Response rate to the follow-up survey according to medical history and current treatments for selected conditions at baseline (Figs. 5 and 6)

Both Aboriginal and non-Aboriginal people who had ever been diagnosed with diabetes were less likely to participate in the follow-up study (0.76, 0.58–0.98 [Aboriginal] vs 0.90, 0.89–0.92 [non-Aboriginal]); this association persisted in the fully adjusted model (Additional file 1: Table S4). Only 35 % of Aboriginal people who had ever been diagnosed with diabetes, participated in the study. Those with severe physical functional limitations were significantly less likely to participate compared to those with no limitations (0.77, 0.75–0.79 [non-Aboriginal]); this association was slightly attenuated in the fully adjusted model (0.82, 0.80–0.84 [non-Aboriginal]). Similarly, those needing help with daily tasks were also less likely to participate in the study compared to those who did not need help (0.71, 0.69–0.74 [non-Aboriginal]), even following further adjustment for formal education and income level (0.76, 0.74–0.79 [non-Aboriginal]). Among current treatments for selected conditions at baseline, Aboriginal and non-Aboriginal individuals who were being treated for heart attack/angina (0.43, 0.21–0.88 [Aboriginal]; 0.92, 0.88–0.95 [non-Aboriginal]) were less likely to participate; this association persisted in the fully adjusted model (0.45, 0.22–0.92 [Aboriginal]; 0.94 (0.91–0.97) [non-aboriginal) (Additional file 1: Table S5). There was a significant statistical interaction in the relationship between study participation and current treatment for heart attack/angina such that Aboriginal participants were even less likely to participate compared to non-Aboriginal people (*χ*^2^ = 6.56; *P* = 0.01). Those participants who were not undergoing treatment for any of the conditions listed were significantly more likely to participate compared to those who were currently undergoing treatment (1.21, 1.02–1.43 [Aboriginal]; 1.06, 1.05–1.07 [non-Aboriginal]); adjusting for formal educational qualifications and household income attenuated this relationship among both Aboriginal (1.11, 0.94–1.30) and non-Aboriginal (1.03, 1.02–1.04) participants (Additional file 1: Table S5).

**Fig. 5:**
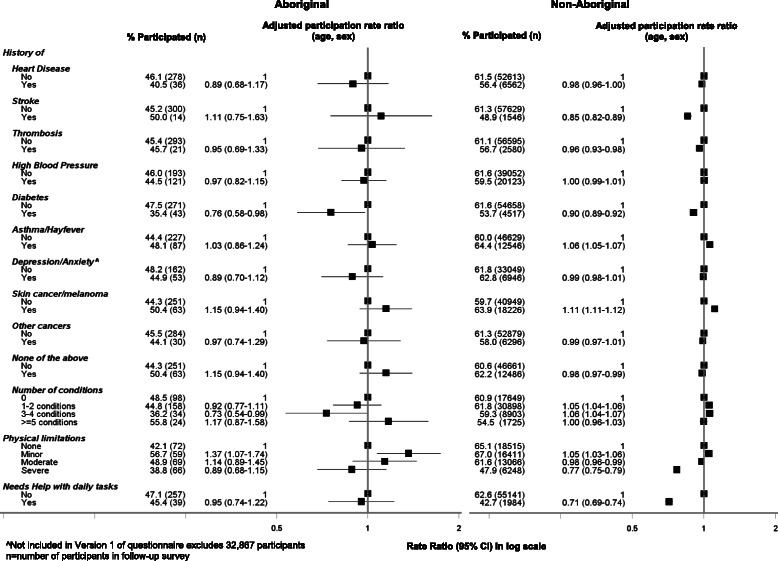
Follow-up participation in the 45 and Up Study among Aboriginal and non-Aboriginal individuals by medical history at baseline

**Fig. 6:**
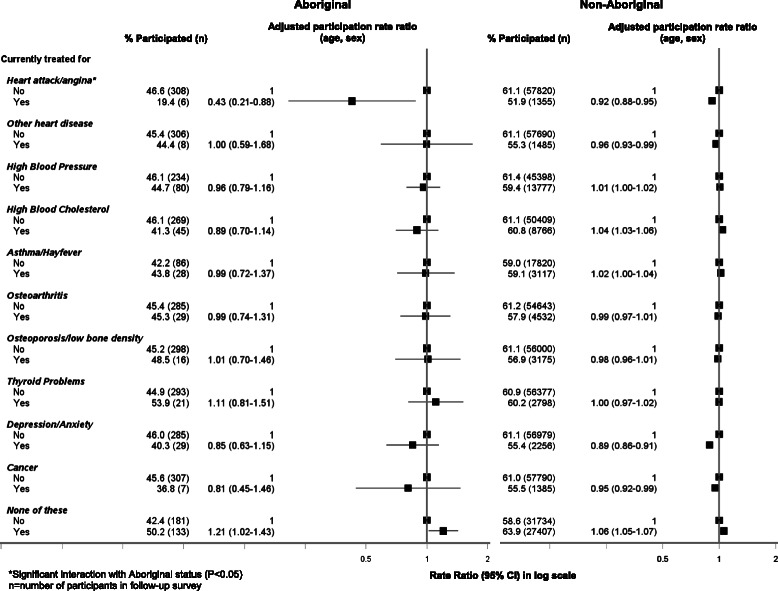
Follow-up participation in the 45 and Up Study among Aboriginal and non-Aboriginal individuals by current treatments for selected medical conditions at baseline

## Discussion

Relatively large numbers of Aboriginal and non-Aboriginal people participated in the first follow-up to the 45 and Up Study. Individuals who responded to the follow-up survey were more likely than non-responders to have tertiary qualifications, earning a higher income and residing in less disadvantaged areas. Individuals who reported being regular smokers at baseline and participants with poor self-rated health/quality of life, high levels of psychological distress and those with fewer social contacts at baseline were significantly less likely to participate in the follow-up survey. Those participants who were being treated for medical conditions at baseline were less likely to respond to the follow-up survey compared to those who were not. Overall, Aboriginal people were less likely to respond to the follow-up study compared to non-Aboriginal people, particularly those participants earning a low income and participants who reported being regular smokers at baseline.

Our finding that almost half of the Aboriginal invitees to the follow-up study participated in the postal survey is encouraging and provides evidence to show that postal surveys can be used to undertake follow-up studies among Aboriginal people. A study, recently published by Marin and colleagues, on obtaining a representative sample for the South Australian Aboriginal population-based health survey [28] reported a 57.7 % response rate; that study used a variety of recruitment strategies and face-to-face interviews for data collection. Reasons for non-participation included: refusals (19.4 %), unavailable for interview (19.5 %), illness/incapability to undertake interview or having moved house since first contact (3.3 %). Previous longitudinal studies that have compared attrition rates among Aboriginal versus non-Aboriginal participants have also shown greater attrition among Aboriginal participants [13–15]. For example, in the Household, Income and Labour Dynamics (HILDA) survey it has been reported that the attrition rate of Aboriginal participants from Wave 1 to Wave 2 was significantly greater compared to non-Aboriginal participants (20 % versus 13 %) [13]; loss to follow-up among Aboriginal people was mostly due to difficulties encountered in contacting the household. In the current study, even after taking into account socio-demographic factors, remoteness of residence, smoking status and number of medical conditions, Aboriginal people were still less likely to participate in the follow-up survey which suggests that other factors not captured in this study (such as cultural differences and opinions on health research) may be related to the lower participation rate.

In the current study, we found that although the response rates were lower among Aboriginal people, the socio-demographic and health-related correlates of participation in follow-up were similar between Aboriginal and non-Aboriginal people. Although studies to examine such associations have not been undertaken in Aboriginal population groups, in Australia or internationally, a Danish study has also suggested that although barriers to participation were relevant to the general population, ethnic minorities may be more exposed to those barriers compared to the general population [29].

The finding that responders of follow-up studies are more socio-economically advantaged compared to non-responders has been shown in a number of previous studies [30–32]. It has been previously suggested that higher education is related to a greater understanding and interest in research studies [33]. Increased participation rates in follow-up studies and in health surveys in general among those socially advantaged may also be associated with higher levels of health literacy. In the current study, a statistically significant interaction with Aboriginal status was found in the association between annual household income and participation in the follow-up survey. This suggests that cohort retention among Aboriginal people who are on a low income may be more difficult compared to non-Aboriginal people.

It is well known that people with a greater level of health risk factors such as tobacco smoking, drug and alcohol abuse are less likely to participate in research studies and also more likely to be lost to follow-up in longitudinal studies [34, 35]. Accordingly, in this study, amongst all the health behaviours examined, we found current smoking to be significantly associated with non-participation in the follow-up study; particularly among the Aboriginal cohort. In general, it is possible that current smokers have a lower interest in aspects of health, including health research compared to non-smokers.

The 'healthy cohort effect' is a well-known concept that has been reported in a number of previous studies [34, 36, 37]; the findings of this study also suggest that the individuals in the cohort retained in the follow-up study are in general physically and mentally healthier. Indeed, participants with a lower self-rated health and quality of life and increased levels of psychological distress were 30–40 % less likely to respond to the follow-up survey. In relation to this finding, we also report that responders to the follow-up survey were significantly less likely to have ever been diagnosed with chronic diseases such as diabetes and stroke, less likely to be currently undergoing medical treatments and less likely to have physical functional limitations. It can be speculated that the reasons for attrition among those suffering from chronic disease and disability could include: difficulty in being contacted due to hospitalisation; difficulty in completing the questionnaire; a need to prioritise things other than participating in research; and feeling disengaged due to mental health problems.

Participation in the follow up survey among those people who had less social support (fewer social contacts or single/not-partnered marital status) was significantly lower compared to those with greater social support. Although the exact reasons for this findings is unclear from this study, previous research has also shown associations between greater social support and positive health behaviours such as participating in cardiovascular disease risk screening [38].

The role of formal educational qualifications and income level as mediating factors was also examined. The association between socio-demographic factors and health with participation in the follow-up survey persisted in the fully adjusted model among both Aboriginal and non-Aboriginal participants. However, a number of the associations were found to be attenuated in the fully adjusted model, suggesting that lower levels of formal education and income level are contributing to a small proportion of the observed associations.

This is one of the few studies to date to have examined the factors associated with participation in follow up surveys, particularly among Aboriginal people. However, one of the main limitations of the current study is that the reasons for non-response to the follow up study is not known, which would have provided greater information to understand patterns of non-response. It is also important to note that due to the relatively small sample size in the Aboriginal group compared to the non-Aboriginal group, some of the associations observed may be prone to error and should be interpreted with caution, particularly where the number of participants in the follow-up survey in the specific category was less than ten [39].

## Conclusions

The findings of this study are important for future analyses and interpretation of longitudinal data from Aboriginal and non-Aboriginal participants in the 45 and Up cohort, as well as follow-up studies more broadly. Importantly, the results show that follow-up studies among Aboriginal participants can be undertaken through postal surveys. Although loss to follow-up was greater among Aboriginal people (even after taking into account age, sex, annual household income, remoteness of residence, smoking status and number of medical conditions), the factors related to non-participation in the follow-up survey were similar between Aboriginal and non-Aboriginal people which included: disadvantage, ill-health and health risk factors. Aboriginal participants on a low annual household income and those who were current regular smokers had a greater likelihood of non-participation compared to non-Aboriginal participants. In future studies, it is important to identify the barriers to participation among hard-to-reach population groups and devise strategies to minimise attrition.

## Consent for publication

Not applicable.

## Availability of data and materials

Due to ethical restrictions, the authors are unable to make the raw data set used for this manuscript publicly available. Readers can visit the 45 and Up study website (https://www.saxinstitute.org.au/our-work/45-up-study/) for more information about how to request the raw data, or they can contact either Sandra Eades (Sandra.Eades@bakeridi.edu.au) or Professor Emily Banks (Emily.Banks@anu.edu.au).

## Additional file

Additional file 1: Table S1. Follow-up participation in the 45 and Up Study among Aboriginal and non-Aboriginal individuals by socio-demographic factors at baseline. **Table S2.** Follow-up participation in the 45 and Up Study among Aboriginal and non-Aboriginal individuals by health behaviours at baseline. **Table S3.** Follow-up participation in the 45 and Up Study among Aboriginal and non-Aboriginal individuals by psychosocial factors at baseline. **Table S4.** Follow-up participation in the 45 and Up Study among Aboriginal and non-Aboriginal individuals by medical history at baseline. **Table S5.** Follow-up participation in the 45 and Up Study among Aboriginal and non-Aboriginal individuals by current treatments for selected conditions at baseline. (DOCX 39.8 kb)

## Abbreviations

ARIA+: Accessibility Remoteness Index of Australia Plus
BMI: body mass index
IRSD: Index of Relative Socio-economic Disadvantage
NSW: New South Wales
PRR: participation rate ratio
WHO: World Health Organisation
