# Supplementary material for: Factors relating to participation in follow-up to the 45 and up study in Aboriginal and non-Aboriginal individuals
Source: BMC Med Res Methodol. 2016 May 11;16:53. doi: 10.1186/s12874-016-0155-x (PMC4865025; doi:10.1186/s12874-016-0155-x)
Supplement: Additional file 1: Table S1. — Follow-up participation in the 45 and Up Study among Aboriginal and non-Aboriginal individuals by socio-demographic factors at baseline. Table S2. Follow-up participation in the 45 and Up Study among Aboriginal and non-Aboriginal individuals by health behaviours at baseline. Table S3. Follow-up participation in the 45 and Up Study among Aboriginal and non-Aboriginal individuals by psychosocial factors at baseline. Table S4. Follow-up participation in the 45 and Up Study among Aboriginal and non-Aboriginal individuals by medical history at baseline. Table S5. Follow-up participation in the 45 and Up Study among Aboriginal and non-Aboriginal individuals by current treatments for selected conditions at baseline. (DOCX 39.8 kb) [file 12874_2016_155_MOESM1_ESM.docx]

**Additional Table 1. Follow-up participation in the 45 and Up Study among Aboriginal and non-Aboriginal individuals by socio-demographic factors at baseline**

|  | **ABORIGINAL** |  | **NON-ABORIGINAL** |  |
| --- | --- | --- | --- | --- |
| **VARIABLE** | **Age, Sex (where appropriate)** | **Age, Sex, Income, Education (where appropriate)** | **Age, Sex (where appropriate)** | **Age, Sex, Income, Education (where appropriate)** |
| **SEX** |  |  |  |  |
| Male | 1.0 | 1.0 | 1.0 | 1.0 |
| Female | 1.20 (1.01-1.43) | 1.19 (1.00-1.40) | 1.03 (1.02-1.04) | 1.08 (1.07-1.09) |
|  |  |  |  |  |
| **AGE IN YEARS** |  |  |  |  |
| 45-49 | 1.0 | 1.0 | 1.0 | 1.0 |
| 50-59 | 1.29 (1.01-1.64) | 1.23 (0.98-1.55) | 1.03 (1.02-1.05) | 1.06 (1.05-1.08) |
| 60-69 | 1.33 (1.02-1.72) | 1.47 (1.15-1.89) | 1.05 (1.03-1.06) | 1.14 (1.12-1.16) |
| 70+ | 1.10 (0.79-1.55) | 1.32 (0.94-1.84) | 0.84 (0.83-0.86) | 0.99 (0.97-1.00) |
|  |  |  |  |  |
| **EDUCATION** |  |  |  |  |
| None | 1.0 | 1.0 | 1.0 | 1.0 |
| School | 1.12 (0.88-1.42) | 1.05 (1.07-1.66) | 1.23 (1.21-1.26) | 1.19 (1.17-1.22) |
| Technical | 1.48 (1.19-1.85) | 1.34 (0.97-1.59) | 1.34 (1.31-1.36) | 1.28 (1.25-1.30) |
| Uni | 1.58 (1.25-2.01) | 1.24 (0.97-1.59) | 1.49 (1.46-1.52) | 1.38 (1.35-1.41) |
|  |  |  |  |  |
| **MARITAL** |  |  |  |  |
| Married | 1.0 | 1.0 | 1.0 | 1.0 |
| Single | 0.68 (0.50-0.94) | 0.73 (0.54-1.00) | 0.90 (0.88-0.93) | 0.93 (0.91-0.95) |
| Widowed | 0.65 (0.44-0.96) | 0.78 (0.53-1.13) | 0.88 (0.86-0.90) | 0.90 (0.88-0.92) |
| Divorced/Separated | 0.92 (0.74-1.14) | 1.01 (0.81-1.27) | 0.91 (0.90-0.93) | 0.93 (0.91-0.94) |
|  |  |  |  |  |
| **WORK STATUS*** |  |  |  |  |
| Paid work | 1.0 | 1.0 | 1.0 | 1.0 |
| Home/family | 0.84 (0.59-1.20) | 1.05 (0.73-1.52) | 0.96 (0.94-0.98) | 1.04 (1.02-1.06) |
| Retired | 0.93 (0.70-1.23) | 1.10 (0.86-1.41) | 1.04 (1.02-1.05) | 1.11 (1.09-1.12) |
| Disabled/sick/  unemployed | 0.72 (0.54-0.95) | 0.95 (0.71-1.28) | 0.76 (0.74-0.79) | 0.89 (0.87-0.92) |
| Other | 1.12 (0.91-1.39) | 1.25 (1.02-1.55) | 1.01 (1.00-1.02) | 1.04 (1.03-1.06) |
|  |  |  |  |  |
| **PRIVATE HEALTH INSURANCE** |  |  |  |  |
| Private | 1.0 | 1.0 | 1.0 | 1.0 |
| DVA Health Care card^ | - | - | 0.87 (0.83-0.90) | 0.91 (0.87-0.94) |
| Healthcare card | 0.65 (0.54-0.78) | 0.77 (0.60-1.01) | 0.87 (0.86-0.88) | 0.96 (0.95-0.97) |
| None | 0.75 (0.62-0.92) | 0.86 (0.70-1.07) | 0.86 (0.85-0.87) | 0.91 (0.89-0.92) |
|  |  |  |  |  |
| **ANNUAL HOUSEHOLD INCOME** |  |  |  |  |
| <$20,000 | 1.0 | 1.0 | 1.0 | 1.0 |
| $20,000-$39,000 | 1.05 (0.83-1.34) | 1.06 (0.82-1.36) | 1.18 (1.16-1.20) | 1.16 (1.14-1.18) |
| $40,000-$69,000 | 1.35 (1.08-1.69) | 1.30 (1.03-1.64) | 1.22 (1.20-1.24) | 1.20 (1.18-1.22) |
| >$70,000 | 1.64 (1.32-2.03) | 1.58 (1.25-2.00) | 1.24 (1.22-1.26) | 1.22 (1.19-1.24) |
|  |  |  |  |  |
| **ARIA** |  |  |  |  |
| Major city | 1.0 | 1.0 | 1.0 | 1.0 |
| Inner regional | 1.35 (1.11-1.64) | 1.38 (1.16-1.64) | 1.06 (1.05-1.07) | 1.07 (1.06-1.09) |
| Outer regional | 0.99 (0.78-1.25) | 1.09 (0.88-1.37) | 1.05 (1.04-1.07) | 1.08 (1.07-1.10) |
| Remote/very remote | 0.75 (0.49-1.16) | 0.87 (0.58-1.32) | 0.97 (0.93-1.01) | 1.03 (0.99-1.06) |
|  |  |  |  |  |
| **IRSD** |  |  |  |  |
| 1 (most disadvantaged) | 1.0 | 1.0 | 1.0 | 1.0 |
| 2 | 1.09 (0.87-1.36) | 1.11 (0.90-1.36) | 1.04 (1.03-1.06) | 1.02 (1.00-1.03) |
| 3 | 1.09 (0.86-1.39) | 1.12 (0.89-1.41) | 1.04 (1.03-1.06) | 1.01 (0.99-1.02) |
| 4 | 1.24 (0.98-1.58) | 1.23 (0.99-1.53) | 1.05 (1.03-1.07) | 1.00 (0.98-1.01) |
| 5 (least disadvantaged) | 1.40 (1.06-1.86) | 1.18 (0.90-1.54) | 1.09 (1.07-1.10) | 0.99 (0.98-1.01) |

*Note: Convergence of the fully adjusted model (age, sex, education, income) for the variable ‘Work status’ among Aboriginal participants is questionable.

^Rate ratio not calculated due to small number of Aboriginal participants (<5)

**Additional Table 2. Follow-up participation in the 45 and Up Study among Aboriginal and non-Aboriginal individuals by health behaviours at baseline**

|  | **ABORIGINAL** | | **NON-ABORIGINAL** | |
| --- | --- | --- | --- | --- |
|  | **Adjusted prevalence rate ratios** | | **Adjusted prevalence rate ratios** | |
| **VARIABLE** | **Age, Sex** | **Age, Sex, Income, Education** | **Age, Sex** | **Age, Sex, Income, Education** |
| **SMOKING STATUS** |  |  |  |  |
| Never smoker | 1.0 | 1.0 | 1.0 | 1.0 |
| Former smoker | 0.95 (0.80-1.13) | 0.96 (0.82-1.12) | 0.96 (0.95-0.97) | 0.97 (0.96-0.98) |
| Current smoker | 0.55 (0.42-0.72) | 0.61 (0.47-0.79) | 0.76 (0.74-0.77) | 0.81 (0.79-0.83) |
|  |  |  |  |  |
| **ALCOHOL INTAKE** |  |  |  |  |
| 0 | 1.0 |  | 1.0 | 1.0 |
| 1-7 | 1.16 (0.96-1.41) | 1.07 (0.90-1.27) | 1.11 (1.09-1.12) | 1.06 (1.05-1.07) |
| 8+ | 1.02 (0.82-1.26) | 0.97 (0.80-1.19) | 1.12 (1.10-1.13) | 1.07 (1.05-1.08) |
|  |  |  |  |  |
| **BMI*** |  |  |  |  |
| Underweight^ | - | - | 0.87 (0.83-0.92) | 0.89 (0.85-0.94) |
| Healthy weight | 1.0 | 1.0 | 1.0 | 1.0 |
| Overweight | 1.14 (0.91-1.41) | 1.10 (0.92-1.31) | 1.00 (0.99-1.02) | 1.01 (1.00-1.03) |
| Obese | 0.94 (0.75-1.18) | 0.98 (0.82-1.17) | 0.94 (0.93-0.96) | 0.98 (0.96-0.99) |
|  |  |  |  |  |
| **SUFFICIENT PA** |  |  |  |  |
| Yes | 1.0 | 1.0 | 1.0 | 1.0 |
| No | 0.84 (0.71-1.00) | 0.91 (0.78-1.07) | 0.86 (0.85-0.87) | 0.89 (0.88-0.90) |
|  |  |  |  |  |
| **SUFFICIENT VEGETABLE INTAKE** |  |  |  |  |
| Yes | 1.0 | 1.0 | 1.0 | 1.0 |
| No | 0.99 (0.83-1.18) | 0.91 (0.78-1.07) | 0.97 (0.96-0.98) | 0.96 (0.95-0.97) |
|  |  |  |  |  |
| **SUFFICIENT FRUIT INTAKE** |  |  |  |  |
| Yes | 1.0 | 1.0 | 1.0 | 1.0 |
| No | 0.86 (0.70-1.05) | 0.91 (0.75-1.10) | 0.94 (0.93-0.95) | 0.96 (0.95-0.97) |
|  |  |  |  |  |
| **SCREEN TIME (HRS/DAY)** |  |  |  |  |
| 0-3 | 1.0 | 1.0 | 1.0 | 1.0 |
| 4-6 | 1.01 (0.84-1.22) | 1.01 (0.85-1.20) | 1.01 (1.00-1.02) | 1.02 (1.01-1.03) |
| >=7 | 1.10 (0.87-1.39) | 1.04 (0.84-1.29) | 1.01 (1.00-1.03) | 1.00 (0.98-1.01) |
|  |  |  |  |  |
| **SITTING TIME (HRS/DAY)** |  |  |  |  |
| 0-3 | 1.0 | 1.0 | 1.0 | 1.0 |
| 4-6 | 1.11 (0.90-1.37) | 1.07 (0.87-1.30) | 1.05 (1.04-1.06) | 1.04 (1.03-1.05) |
| >=7 | 1.14 (0.90-1.44) | 1.03 (0.83-1.29) | 1.06 (1.05-1.08) | 1.03 (1.02-1.04) |

*Note: Convergence of the fully adjusted model (age, sex, income, education) for the variable ‘BMI’ among Aboriginal participants is questionable.

^Rate ratio not calculated due to small number of Aboriginal participants (n<5)

**Additional Table 3. Follow-up participation in the 45 and Up Study among Aboriginal and non-Aboriginal individuals by psychosocial factors at baseline**

|  | **ABORIGINAL** | | **NON-ABORIGINAL** | |
| --- | --- | --- | --- | --- |
|  | **Adjusted prevalence rate ratios** | | **Adjusted prevalence rate ratios** | |
| **VARIABLE** | **Age, Sex** | **Age, Sex, Education, Income** | **Age, Sex** | **Age, Sex, Income, Education** |
| **CARER STATUS** |  |  |  |  |
| None | 1.0 | 1.0 | 1.0 | 1.0 |
| Part-time | 1.09 (0.83-1.43) | 1.14 (0.88-1.48) | 1.01 (0.99-1.03) | 1.00 (0.99-1.02) |
| Full-time | 0.78 (0.54-1.12) | 0.83 (0.58-1.18) | 0.90 (0.87-0.93) | 0.95 (0.92-0.98) |
|  |  |  |  |  |
| **SOCIAL CONTACTS** |  |  |  |  |
| None | 1.0 | 1.0 | 1.0 | 1.0 |
| 1-3 people | 0.93 (0.66-1.30) | 0.87 (0.63-1.21) | 1.04 (1.02-1.07) | 1.03 (1.00-1.05) |
| 4-6 people | 1.11 (0.80-1.54) | 0.97 (0.70-1.33) | 1.12 (1.09-1.15) | 1.09 (1.07-1.12) |
| >= 7 people | 1.21 (0.88-1.66) | 1.04 (0.76-1.44) | 1.16 (1.14-1.19) | 1.12 (1.09-1.15) |
|  |  |  |  |  |
| **SELF-RATED HEALTH*** |  |  |  |  |
| Excellent/very good | 1.0 | 1.0 | 1.0 | 1.0 |
| Good/fair | 0.75 (0.63-0.89) | 0.84 (0.73-0.98) | 0.85 (0.84-0.86) | 0.89 (0.88-0.90) |
| Poor | 0.68 (0.47-0.99) | 0.82 (0.60-1.12) | 0.65 (0.61-0.69) | 0.71 (0.67-0.76) |
|  |  |  |  |  |
| **SELF-RATED QUALITY OF LIFE*** |  |  |  |  |
| Excellent/very good | 1.0 | 1.0 | 1.0 | 1.0 |
| Good/fair | 0.70 (0.59-0.83) | 0.82 (0.71-0.96) | 0.82 (0.81-0.83) | 0.86 (0.85-0.87) |
| Poor | 0.63 (0.41-0.97) | 0.83 (0.58-1.21) | 0.61 (0.57-0.66) | 0.68 (0.63-0.72) |
|  |  |  |  |  |
| **PSYCHOLOGICAL DISTRESS** |  |  |  |  |
| Low | 1.0 | 1.0 | 1.0 | 1.0 |
| Moderate | 0.96 (0.77-1.20) | 1.06 (0.85-1.31) | 0.93 (0.91-0.94) | 0.94 (0.93-0.95) |
| High | 0.83 (0.61-1.13) | 1.03 (0.75-1.40) | 0.83 (0.80-0.85) | 0.87 (0.84-0.89) |
| Very high | 0.77 (0.54-1.10) | 0.97 (0.68-1.39) | 0.71 (0.68-0.75) | 0.78 (0.74-0.79) |

*Note: Convergence of the fully adjusted model (age, sex, education, income) for the variables ‘Self-rated health’ and ‘Self-rated quality of life’ among Aboriginal participants is questionable.

**Additional Table 4. Follow-up participation in the 45 and Up Study among Aboriginal and non-Aboriginal individuals by medical history at baseline**

|  | **ABORIGINAL** | | **NON-ABORIGINAL** | |
| --- | --- | --- | --- | --- |
|  | **Adjusted prevalence rate ratios** | | **Adjusted prevalence rate ratios** | |
| **VARIABLE** | **Age, Sex** | **Age, Sex, Income, Education** | **Age, Sex** | **Age, Sex, Income, Education** |
| **Heart disease** |  |  |  |  |
| No | 1.0 | 1.0 | 1.0 | 1.0 |
| Yes | 0.89 (0.68-1.17) | 0.98 (0.97-1.00) | 0.98 (0.96-1.00) | 0.92 (0.71-1.18) |
|  |  |  |  |  |
| **Stroke** |  |  |  |  |
| No | 1.0 | 1.0 | 1.0 | 1.0 |
| Yes | 1.11 (0.75-1.63) | 1.02 (0.71-1.48) | 0.85 (0.82-0.89) | 0.88 (0.85-0.91) |
|  |  |  |  |  |
| **Thrombosis** |  |  |  |  |
| No | 1.0 | 1.0 | 1.0 | 1.0 |
| Yes | 0.95 (0.69-1.33) | 1.14 (0.83-1.56) | 0.96 (0.93-0.98) | 0.97 (0.95-1.00) |
|  |  |  |  |  |
| **High blood pressure** |  |  |  |  |
| No | 1.0 | 1.0 | 1.0 | 1.0 |
| Yes | 0.97 (0.82-1.15) | 0.97 (0.81-1.15) | 1.00 (0.99-1.01) | 1.01 (1.00-1.03) |
|  |  |  |  |  |
| **Diabetes** |  |  |  |  |
| No | 1.0 | 1.0 | 1.0 | 1.0 |
| Yes | 0.76 (0.58-0.98) | 0.77 (0.61-0.98) | 0.90 (0.89-0.92) | 0.94 (0.92-0.95) |
|  |  |  |  |  |
| **Asthma/hayfever** |  |  |  |  |
| No | 1.0 | 1.0 | 1.0 | 1.0 |
| Yes | 1.03 (0.86-1.24) | 1.01 (0.85-1.19) | 1.06 (1.05-1.07) | 1.04 (1.03-1.05) |
|  |  |  |  |  |
| **Depression/anxiety** |  |  |  |  |
| No | 1.0 |  | 1.0 |  |
| Yes | 0.89 (0.70-1.12) | 1.01 (0.85-1.19) | 0.99 (0.98-1.01) | 1.04 (1.03-1.05) |
|  |  |  |  |  |
| **Skin cancer/melanoma** |  |  |  |  |
| No | 1.0 | 1.0 | 1.0 | 1.0 |
| Yes | 1.15 (0.94-1.40) | 1.09 (0.91-1.31) | 1.11 (1.11-1.12) | 1.08 (1.07-1.10) |
|  |  |  |  |  |
| **Other cancers** |  |  |  |  |
| No | 1.0 | 1.0 | 1.0 | 1.0 |
| Yes | 0.97 (0.74-1.29) | 1.00 (0.76-1.30) | 0.99 (0.97-1.01) | 0.99 (0.97-1.00) |
|  |  |  |  |  |
| **None of the above** |  |  |  |  |
| No | 1.0 | 1.0 | 1.0 | 1.0 |
| Yes | 1.15 (0.94-1.40) | 1.03 (1.02-1.04) | 0.98 (0.97-0.99) | 1.10 (0.94-1.30) |
|  |  |  |  |  |
| **Number of conditions** |  |  |  |  |
| 0 | 1.0 |  | 1.0 | 1.0 |
| 1-2 conditions | 0.92 (0.77-1.11) | 0.97 (0.82-1.15) | 1.05 (1.04-1.06) | 1.04 (1.03-1.05) |
| 3-4 conditions | 0.73 (0.54-0.99) | 0.79 (0.59-1.07) | 1.06 (1.04-1.07) | 1.06 (1.04-1.07) |
| >=5 conditions | 1.17 (0.87-1.58) | 1.26 (0.94-1.69) | 1.00 (0.96-1.03) | 1.01 (0.98-1.04) |
|  |  |  |  |  |
| **Physical limitations*** |  |  |  |  |
| None | 1.0 | 1.0 | 1.0 | 1.0 |
| Minor | 1.37 (1.07-1.74) | 1.18 (0.95-1.47) | 1.05 (1.03-1.06) | 1.04 (1.03-1.05) |
| Moderate | 1.14 (0.89-1.45) | 1.02 (0.83-1.26) | 0.98 (0.96-0.99) | 0.99 (0.98-1.01) |
| Severe | 0.89 (0.68-1.15) | 0.94 (0.75-1.18) | 0.77 (0.75-0.79) | 0.82 (0.80-0.84) |
|  |  |  |  |  |
| **Needs help with daily tasks** |  |  |  |  |
| No | 1.0 | 1.0 | 1.0 | 1.0 |
| Yes | 0.95 (0.74-1.22) | 1.11 (0.86-1.42) | 0.71 (0.69-0.74) | 0.76 (0.74-0.79) |

*Note: Convergence of the fully adjusted model (age, sex, education, income) for the variable ‘Physical Limitations’ among Aboriginal participants is questionable.

**Additional Table 5.** **Follow-up participation in the 45 and Up Study among Aboriginal and non-Aboriginal individuals by current treatments for selected conditions at baseline**

|  | **ABORIGINAL** | | **NON-ABORIGINAL** | |
| --- | --- | --- | --- | --- |
|  | **Adjusted prevalence rate ratios** | | **Adjusted prevalence rate ratios** | |
| **VARIABLE** | **Age, Sex** | **Age, Sex, Income, Education** | **Age, Sex** | **Age, Sex, Income, Education** |
| **Heart Attack/angina** |  |  |  |  |
| No | 1.0 | 1.0 | 1.0 | 1.0 |
| Yes | 0.43 (0.21-0.88) | 0.45 (0.22-0.92) | 0.92 (0.88-0.95) | 0.94 (0.91-0.97) |
|  |  |  |  |  |
| **Other heart disease** |  |  |  |  |
| No | 1.0 | 1.0 | 1.0 | 1.0 |
| Yes | 1.00 (0.59-1.68) | 0.67 (0.42-1.06) | 0.96 (0.93-0.99) | 0.95 (0.93-0.98) |
|  |  |  |  |  |
| **High Blood Pressure** |  |  |  |  |
| No | 1.0 | 1.0 | 1.0 | 1.0 |
| Yes | 0.96 (0.79-1.16) | 0.97 (0.81-1.15) | 1.01 (1.00-1.02) | 1.01 (1.00-1.03) |
|  |  |  |  |  |
| **High blood cholesterol** |  |  |  |  |
| No | 1.0 | 1.0 | 1.0 | 1.0 |
| Yes | 0.89 (0.70-1.14) | 0.90 (0.72-1.13) | 1.04 (1.03-1.06) | 1.05 (1.03-1.06) |
|  |  |  |  |  |
| **Asthma/Hayfever** |  |  |  |  |
| No | 1.0 | 1.0 | 1.0 | 1.0 |
| Yes | 0.99 (0.72-1.37) | 1.04 (0.77-1.40) | 1.02 (1.00-1.04) | 1.02 (1.00-1.05) |
|  |  |  |  |  |
| **Osteoarthritis** |  |  |  |  |
| No | 1.0 | 1.0 | 1.0 | 1.0 |
| Yes | 0.99 (0.74-1.31) | 1.11 (0.84-1.48) | 0.99 (0.97-1.01) | 1.00 (0.98-1.02) |
|  |  |  |  |  |
| **Osteoporosis/low bone density** |  |  |  |  |
| No | 1.0 | 1.0 | 1.0 | 1.0 |
| Yes | 1.01 (0.70-1.46) | 1.04 (0.73-1.47) | 0.98 (0.96-1.01) | 0.99 (0.97-1.01) |
|  |  |  |  |  |
| **Thyroid problems** |  |  |  |  |
| No | 1.0 | 1.0 | 1.0 | 1.0 |
| Yes | 1.11 (0.81-1.51) | 1.05 (0.81-1.36) | 1.00 (0.97-1.02) | 1.00 (0.98-1.03) |
|  |  |  |  |  |
| **Depression/Anxiety** |  |  |  |  |
| No | 1.0 | 1.0 | 1.0 | 1.0 |
| Yes | 0.85 (0.63-1.15) | 0.91 (0.69-1.20) | 0.89 (0.86-0.91) | 0.92 (0.90-0.95) |
|  |  |  |  |  |
| **Cancer** |  |  |  |  |
| No | 1.0 | 1.0 | 1.0 | 1.0 |
| Yes | 0.81 (0.45-1.46) | 0.80 (0.45-1.42) | 0.95 (0.92-0.99) | 0.96 (0.92-0.99) |
|  |  |  |  |  |
| **None of these** |  |  |  |  |
| No | 1.0 | 1.0 | 1.0 | 1.0 |
| Yes | 1.21 (1.02-1.43) | 1.11 (0.94-1.30) | 1.06 (1.05-1.07) | 1.03 (1.02-1.04) |
